# Prevalence of malnutrition and associated factors among hospitalized elderly patients in King Abdulaziz University Hospital, Jeddah, Saudi Arabia

**DOI:** 10.1186/s12877-017-0527-z

**Published:** 2017-07-03

**Authors:** Sami H. Alzahrani, Sultan H. Alamri

**Affiliations:** Family and Community Medicine Department, Faculty of Medicine, King Abdulaziz University, PO Box 80205, Jeddah, 21589 Saudi Arabia

**Keywords:** Malnutrition, Geriatric, Elderly, Hospitalized, Health services

## Abstract

**Background:**

Malnutrition is a nutritional disorder that adversely affects the body from a functional or clinical perspective. It is very often observed in the elderly population. This study aimed to estimate the prevalence of malnutrition among hospitalized elderly patients and its associated factors and outcomes in terms of length of stay and mortality in King Abdulaziz University Hospital, Jeddah, Saudi Arabia.

**Methods:**

In a cross-sectional study, we evaluated the nutritional status of hospitalized elderly patients using the most recent version of the short form of Mini Nutritional Assessment (MNA-SF).

**Results:**

A total of 248 hospitalized patients were included (70.0 ± 7.7 years; 60% female). According to the MNA-SF, a total of 76.6% patients were either malnourished or at risk of malnutrition. Malnourished patients had significantly lower levels of serum albumin (28.2 ± 7.7), hemoglobin (10.5 ± 1.8), and lymphocyte (1.7 ± 0.91). They had increased tendency to stay in the hospital for longer durations (IQR, 5-11 days; median = 7 days) and had a mortality rate of 6.9%.

**Conclusion:**

Malnutrition was highly prevalent among hospitalized elderly and was associated with increased length of stay and mortality.

## Background

Malnutrition is a nutritional disorder that adversely affects the body from a functional or clinical perspective [1]. It is very often observed in the elderly population. A retrospective pooled analysis of elderly people from 12 countries reported that the overall prevalence of malnutrition was approximately 23%. The highest prevalence was observed in rehabilitation settings (50.5%), followed by hospitals (38.7%), nursing homes (13.8%), and communities (5.8%) [2].

Malnutrition is associated with several adverse health outcomes in the elderly, including increased morbidity and mortality, prolonged hospital stays [3], functional impairment [4], poor quality of life [5], increased infections, electrolyte imbalances, anemia, muscle wasting, and fatigue [6, 7]. There is also an association between falls and undernourishment in elderly people who are either hospitalized or living in long-term care facilities [8]. Despite these consequences, several reports have shown that malnutrition remains under-recognized in the hospitalized elderly, where is no standard procedure is available for proper documentation due to insufficient awareness among healthcare professionals [9]. Therefore, an early identification of malnutrition, as well as nutritional intervention, is of considerable importance in helping to delay or prevent associated poor outcomes, [10] This indicates the need of activation/establishing services dedicated to assess the nutritional status of elderly people. In this respect, several screening tools have been developed to identify malnutrition [10]. The Mini Nutritional Assessment (MNA) is an 18-point nutritional screening tool that has gained worldwide acceptance, relying on anthropometrical, medical, lifestyle, dietary, and psychosocial factors [11]. It was designed specifically for use among the elderly population and has been validated in many care settings, including communities, hospitals, and nursing homes [12]. A short form of MNA (MNA-SF) has been developed as a time-saving nutritional screening tool that is easy to use in routine practice with comparable diagnostic accuracy to the full MNA [13]. The most recent version of MNA-SF provides a quick nutritional screening with the option of using calf circumference when BMI is unavailable [14].

The objectives of this study were 1) to estimate the prevalence of malnutrition among elderly population admitted to King Abdulaziz University Hospital (KAUH) in Jeddah, Saudi Arabia using the revised version of Mini Nutritional Assessment (MNA-SF) and 2) to assess relationships between their nutritional status, socio-demographic data, mortality and length of stay (LOS).

## Methods

### Setting and sample

In a cross-sectional study, we used the most recent version of the short form of Mini Nutritional Assessment (MNA-SF) to evaluated the nutritional status of hospitalized elderly patients, it was carried out at King Abdulaziz University Hospital in Jeddah, Saudi Arabia. A tow repeated annual cross-sectional surveys (pseudo-longitudinal study) was carried out to estimate the length of stay, readmission and mortality rates. The sample size was calculated by using the single proportion equation in the Raosoft software package. Considering the frequency of having sufficient knowledge as 50%, accepted margin of error was 5% and the confidence level was 95%. The estimated sample size was 184, it was increased to 248 to compensate for non-responders.

During the month of February 2015, a total of 248 consecutive elderly patients (over 60 years of age) were evaluated by a single investigator within the first 48 h of admission, all patients with limb amputation, with terminal cancer, re-admitted within the study period, suffering from any abnormality that will interfere with accurate measurement of body weight and/or height & Patients (or their relatives) unable or unwilling to give written informed consent were excluded from the study.

### Study tools

Two tools were used to collect the data: 1- First a specially designed questionnaire was used to collect socio-demographic variables including patient’s age, gender, marital status, living arrangement, level of education, income, smoking history, alcohol consumption, chronic illnesses, and polypharmacy.

2- Second: Patient’s nutritional status was evaluated using the most recent version of the short form of Mini Nutritional Assessment (MNA-SF) [13]. This instrument has a comparable sensitivity to the full MNA with the option of measuring calf circumference (CC) when Body Mass Index (BMI) is unavailable. It has a maximum score of 14 and categorizes patients into malnourished (less than 8), at risk for malnutrition (from 8 to 11), or normal nutritional status (more than 11) [14]. This revised MNA-SF has the advantages of fast screening and increased clinical applicability in practice through malnourished category inclusion [14].

All anthropometric data including body mass index (BMI), calf circumference (CC), weight and height were recorded using standard techniques. Biochemical and hematological parameters including hemoglobin (g/L), albumin (g/L), and lymphocyte count (× 10^9/L) were obtained from patient’s chart and recorded for analysis.

Readmission, length of stay and mortality outcomes were determined within one year after discharge.

### Statistical analysis

SPSS version 23.0 was used to manage and analyze the data. Normally distributed variables were expressed as means (standard deviation [SD]), and non-normally distributed variables were presented as medians (IQR). Categorical variables were expressed as numbers and percentages. The chi-square test was used to assess the significance of categorical variables. Binary logistic regression (enter method) was carried out to identify predictors of being categorized as malnourished (BMI and calf circumference, food intake, and mobility, live arrangement), variables excluded from the model were Albumin, Hemoglobin, and Lymphocyte. *P* value ≤0.05 was considered statistically significant. Overall survival rates were investigated using Kaplan-Meier method.

## Results

Based on the study design and assigned population, the mean age of the elderly hospitalized patients was 70.0 ± 7.7 years; it ranged between 60 and 94 years. Females constituted almost two-thirds (60.5%) of them. Most of them were married (85.5%) or widowed (13.3%), and the majority were illiterate (73.4%), with a monthly income of <3000 SR (US $800) (85.9%). The vast majority lived with others (83.1%). The majority of the patients (210, 84.7%) reported that they were diagnosed previously with a chronic disease, of them, there were 143 patients (57.7%) who were diabetic and 132 (53.2%) who were hypertensive in addition to 68 patients (27.4%) with IHD and 60 (24.2%) had dyslipidemia.

Overall, more than one-quarter of the patients 72 (29%) were overtly malnourished (MNA-SF score 0-7 points), while almost one-half 118 (47.6%) were at risk of becoming malnourished (MNA-SF score 8-11 points), making a total of 76.6% who were either malnourished or at nutritional risk, and the rest 58 (23.4%) were within normal.

In order to compare differences within elderly patients according to their nutritional status (as revealed by their MNA-SF scores), Table 1 shows that there was no difference according to gender. Among other demographic and social factors, only living alone showed a significant difference, where it was found that 20% of the malnourished or those at risk of malnourishment were living alone (*p* < 0 .001) (Table 1).

**Table 1:** Nutritional status of geriatric patients according to their demographic characteristics (*n* = 248)

Variable	Normal nutrition, *n* = 58	Malnourished, *n* = 118	at risk of malnutrition, *n* = 72	*p*-value
Demographic/social characteristics:				
Gender, n (%)				
Males	25 (43.1)	45 (38.1)	28 (38.9)	
Females	33 (56.9)	73 (61.9)	44 (61.1)	0.649
Age, mean (SD)	69.1 ± 8.0	69.5 ± 7.2	71.5 ± 8.1	0.138
Living arrangements, n (%)				
Living with others	56 (96.6)	109 (92.4)	43 (59.7)	
Living alone	2 (3.4)	9 (7.6)	29 (40.3)	< 0.001
Monthly income, n (%)				
< US $800	51 (87.9)	95 (80.5)	67 (93.0)	0.459
> US $800	7 (12.1)	23 (19.5)	5 (7.0)	

Data are presented as number & percentage (%) or as mean & standard deviation

Significance between groups was determined using chi-square test for categorical variables and one-way ANOVA for continuous variables

Regarding the anthropometric measures that partly reflect malnutrition, as expected, 6.3% of the malnourished patients were categorized as being underweight compared to 0% of the normally nourished; also, the mean calf circumference was significantly lower among the malnourished patients. The laboratory investigation results showed that the malnourished elderly patients had significantly lower serum albumin levels (28.2 ± 7.7), hemoglobin levels (10.5 ± 1.8), and lymphocyte count (1.7 ± 0.91). Most of the patients 210 (84.7%) reported being previously diagnosed with a chronic disease; of these, 143 patients (57.7%) were diabetic and 132 (53.2%) were hypertensive; while 68 patients (27.4%) had ischemic heart disease (IHD) and 60 (24.2%) had dyslipidemia.

When compared to patients with a normal nutritional status, a significantly higher percentage of malnourished patients experienced either a severe (18.4%) or moderate (45.8%) decrease in food intake. Moreover, a significantly higher percentage of malnourished patients (50%) reported that they could not walk independently and mobilized either with assistance (19.5%), with wheelchairs (10.5%), or were bedbound (20%). Furthermore, malnourished elderly patients were significantly more likely to be readmitted when compared to patients with a normal nutritional status, with a total of (45.3%) being readmitted for an average of 7 days (IQR, 5-11 days). Lastly, out of the 7 reported mortalities, 5 were elderly malnourished patients (Table 2).

**Table 2:** Nutritional status of geriatric patients according to their clinical characteristics and outcome (*n* = 248)

Variable	Normal nutrition, *n* = 58	Malnourished, *n* = 118	at risk of malnutrition, *n* = 72	*p*-value
Anthropometric measures:				
BMI categories, n (%)				
Underweight	0 (0.0)	36 (30.5)	40 (55.6)	
Within normal	6 (10.5)	17 (14.4	5 (6.9)	< 0.001
Overweight	26 (45.6)	30 (25.4)	9 (12.5)	
Obese	25 (43.9)	35 (29.7)	18 (25.0)	
Calf circumference, n (%)				
< 31 cm	7 (12.1)	47 (39.8)	41 (56.9)	< 0.001
> 31 cm	51 (87.9)	71 (60.2)	31 (43.1)	
Clinical characteristics				
Chronic illnesses, n (%)	52 (89.1)	96 (81.4)	62 (86.1)	0.651
Diabetes Mellitus, n (%)				
Yes	42 (72.4)	60 (508)	41 (56.9)	0.107
No	16 (27.6)	58 (49.2)	31 (43.1)	
Albumin, mean (SD)	34.5 (5.5)	29.6 (7.1)	25.9 (8.3)	< 0.001
Hemoglobin, mean (SD)	12.1 (1.3)	11.0 (1.8)	9.7 (1.3)	< 0.001
Lymphocytes mean (SD)	2.2 (0.85)	1.9 (0.88)	1.5 (0.94)	< 0.001
Food intake, n (%)				
Severe decrease in food intake	0 (0.0)	2 (1.7)	33 (45.8)	
Moderate decrease in food intake	7 (12.1)	64 (54.2)	23 (31.9)	< 0.001
No decrease in food intake	51 (87.9)	52 (44.1)	16 (22.3)	
Mobility and walking ability, n(%)				
Can walk without assistance	46 (79.3)	86 (72.9)	24 (33.3)	
Can walk but with assistance	7 (12.1)	14 (11.8)	8 (11.2)	
Moves with a wheelchair	5 (8.6)	8 (6.8)	7 (9.7)	< 0.001
Bedbound	0 (0.0)	10 (8.5)	33 (45.8)	
Readmission, n (%)				
No	45 (77.6)	90 (67.3)	14 (19.4)	
Once	11 (19.0)	28 (23.7)	22 (30.6)	< 0.001
Twice or more	2 (3.4)	0 (0.0)	36 (50.0)	
Length of stay, median (IQR)	4.5 (3-6)	5 (4-7)	12.5 (9-17)	< 0.001

Data are presented as number & percentage (%) or as mean & standard deviation

Significance between groups was determined using chi-square test for categorical variables and one-way ANOVA for continuous variables

Binary logistic regression revealed that the significant independent predictors for being categorized as at nutritional risk or malnourished include a lower BMI and calf circumference, in addition to reduced food intake and mobility (*p* < 0.05). Living alone also showed a borderline level of significance (*p* = 0.082) as an independent predictor (Table 3).

**Table 3:** Predictors of malnutrition among geriatric inpatients

	*p*	Odds Ratio	95% C.I. for Odds Ratio	
			Lower	Upper
BMI	0.000	0.146	0.071	0.287
Calf Circumference	0.016	1.837	1.132	3.072
Mobility	0.002	0.056	0.010	0.346
Food intake	0.000	0.077	0.027	0.219
Living arrangements	0.082	4.856	0.457	4.149
Constant	0.000	9.548		
Cox & Snell R Square = 0.427		Nagelkerke R Square = 0.643		

Binary logistic regression was used (enter method)

**Fig. 1:**
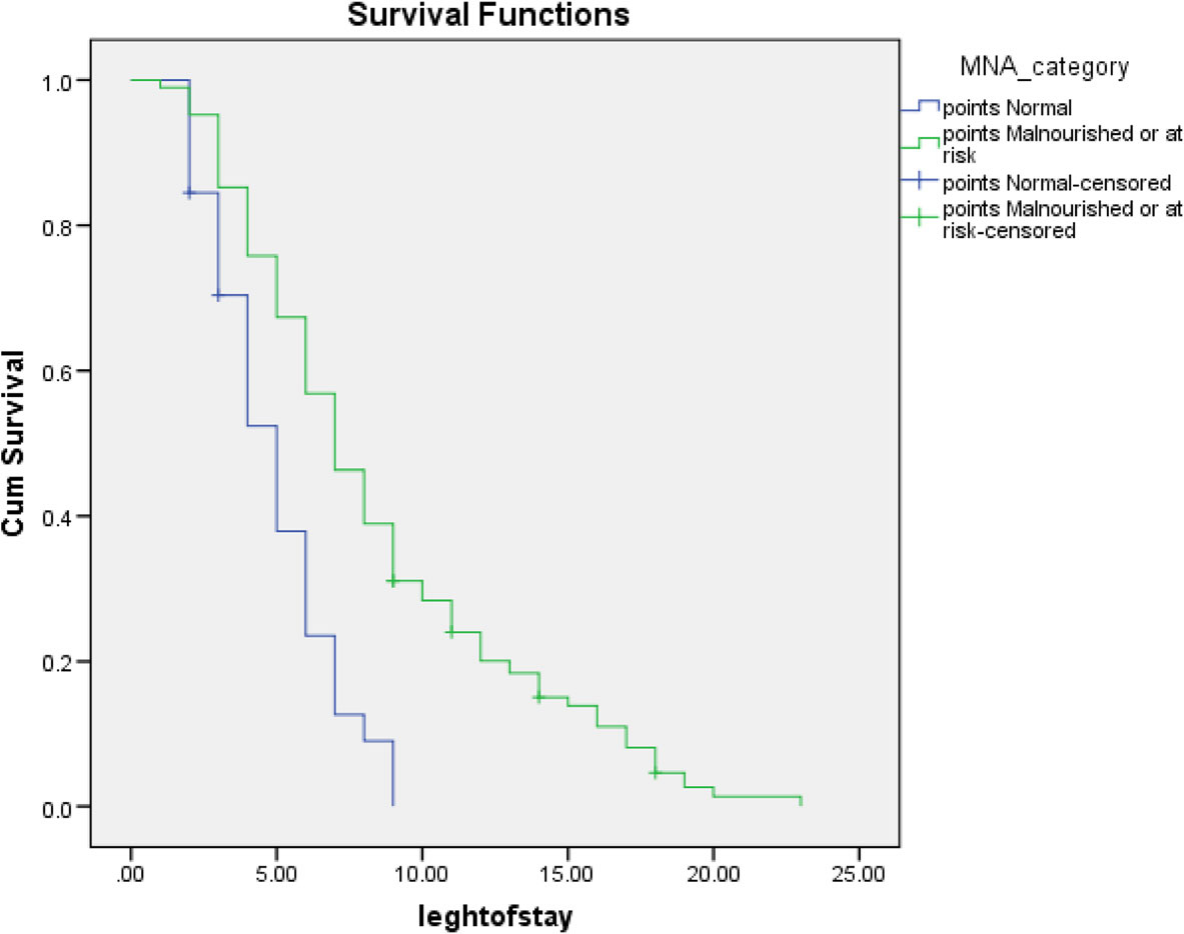
Mortality and survival rates

Regarding mortality and survival rate, the median of the length of stay were 5.0 months (95%CI: 4.2-5.7) among normal nutrition patients and 7.0 months (95%CI: 6.2-7.7) among malnutrition patients (Fig. 1).

## Discussion

As people get older, many changes typically occur that affect nutritional status. Sensory changes, including smell, vision, and taste, affect the appetite in many ways that lead to a diminished dietary intake; these, together with changes in the digestive system that alter absorption and the digestion of food, can lead to malnutrition. The problem of malnutrition becomes more apparent and remarkable when elderly individuals are hospitalized. Hospitalized patients especially the elderly have an increased vulnerability due to their catabolic state, decreased immune system, investigations that require fasting, delay of nutritional support and the presenting disease manifestations. These factors increase the nutritional requirements, resulting in utilization of the nutritional reserves that lead to malnutrition [15].

The worldwide prevalence of malnutrition among hospitalized geriatric patients ranges from 12% to 75% [16]. In our study, the prevalence of poorly nourished patients reached the highest recorded limit, with three-quarters of the hospitalized elderly patients (76.6%) having a poor nutritional status; this was higher than that reported in a multinational study where approximately two-thirds of study participants were determined to be malnourished or at nutritional risk [2]. However, our findings were congruent with the results of another study conducted in Makkah Governorate, Saudi Arabia, which used the Mini Nutritional Assessment (MNA) tool and determined that among the studied patients, (22.6%) were malnourished, (57.8%) were at risk of malnutrition, and (19.6%) were well-nourished [17]. These figures indicate that malnutrition is highly prevalent among hospitalized elderly patients. This high prevalence could be attributed to several factors related to the reasons for hospital admission, including age-related coexisting medical conditions that can result in low food intake [18], polypharmacy that can be an iatrogenic cause of malnutrition [19], exaggerated decline in senses of taste and/or smell with resultant poor appetite [20, 21], and cognitive changes such as dementia [22]. The relatively higher percentage in our patients may additionally indicate poor dietary habits.

Other factors that contribute to malnutrition among elderly individuals are social isolation and financial deprivation [18]. In our study, the percentage of malnourished geriatrics that lived alone was significantly higher than the percentage living with others (*p* < .001). This emphasizes the important role of social support for this age group, as they are often unable to prepare food or even serve themselves [23].

Aging is characterized by the loss of lean muscle mass (sarcopenia), with a noticeable loss of strength, functional decline, and poor endurance [24]. Another common symptom of aging are changes in bone density, which can increase the risk for osteoporosis [25]. These changes progressively limit the mobility of elderly individuals, which makes shopping, preparing food, and even eating more difficult. Limited mobility was apparent in our malnourished geriatric patients, as most of them indicated that they needed assistance with walking, used wheelchairs, or were bedridden.

These factors, in addition to economic factors, may well affect the frequency, quantity, and quality of food intake. In our study, a significantly higher percentage of malnourished patients experienced either severe (18.4%) or moderate (45.8%) decreases in food intake. These findings are comparable to reported findings by Elmadbouly and Abdelhafez [17], who showed that 52.2% of malnourished elderly patients had severe declines in food intake. Similar results were observed by Oliveira et al. [26] and were attributed to anorexia, chewing or swallowing problems and digestion problems. Poverty and cognitive impairment are other issues that may affect eating habits and food choices [27].

Serum proteins have been used as markers of nutrition, with serum albumin being the most widely adopted because of its ability to predict mortality and other outcomes in older people [28]. Reuben et al. [29] reported that geriatrics with albumin <38 g/L had higher hospital resource use. In our study, it was found that malnourished geriatric patients had significantly lower serum albumin levels (28.2 ± 7.7), which could predict overutilization of the hospital resources. This observation is supported by Herrmann et al., who found that low albumin levels in hospitalized geriatrics, were associated with longer periods of hospital stays and increased the risk of being readmitted to the hospital within one year [30].

The MNA-SF has been determined to be a powerful tool for predicting the length of stay of hospitalized elderly patients [31]. In our study, it was found that malnourished patients had a significantly longer length of stay than their well-nourished counterparts, which emphasizes the important role of nutrition in patient recovery [32] and delayed recovery [33]. Therefore, a malnourished elderly patient when admitted to the hospital will have increased risks of more complications than a well-nourished patient with the same medical condition, consequently resulting in a longer hospital stay [34].

For over 7 decades, it has been documented that malnutrition among elderly hospitalized patients influences their mortality rate, which ranges from 20 to 50% [35]. In the current study, 5 out of the 7 malnourished patients (*n* = 72) died, reporting a mortality rate of 6.9%.

There is a limitation of this study represented by small sample size and the heterogeneity of the study group with different diseases at different stages, which potentially could influence the generalizability of our findings.

## Conclusions

Malnutrition was highly prevalent among hospitalized elderly and was associated with increased length of stay and mortality. It is, therefore, essential to assess the nutritional status of elderly patients early in admission and to institute appropriate nutritional therapy to minimize its devastating consequences on the patients and health care system.

## Abbreviations

BMI: Body mass index
CC: Calf circumference
KAUH: King Abdulaziz University Hospital
LOS: Length of stay
MNA: Mini Nutritional Assessment Tool
MNA-SF: Mini Nutritional Assessment Tool - Short Form
